# Cisplatin, Oxaliplatin, and Kiteplatin Subcellular Effects Compared in a Plant Model

**DOI:** 10.3390/ijms18020306

**Published:** 2017-01-31

**Authors:** Paride Papadia, Fabrizio Barozzi, James D. Hoeschele, Gabriella Piro, Nicola Margiotta, Gian-Pietro Di Sansebastiano

**Affiliations:** 1 Department of Biotechnology and Environmental Sciences, University of Salento, via Monteroni-Centro Ecotekne, 73100 Lecce, Italy; paride.papadia@unisalento.it (P.P.); fabrizio.barozzi@unisalento.it (F.B.); gabriella.piro@unisalento.it (G.P.); 2 Department of Chemistry, Eastern Michigan University, Ypsilanti, MI 48197, USA; hoeschel@chemistry.msu.edu; 3 Department of Chemistry, University of Bari Aldo Moro, Via E. Orabona 4, 70125 Bari, Italy

**Keywords:** cytoskeleton, vacuoles, transgenic *Arabidopsis*, cisplatin, kiteplatin, oxaliplatin

## Abstract

The immediate visual comparison of platinum chemotherapeutics’ effects in eukaryotic cells using accessible plant models of transgenic *Arabidopsis thaliana* is reported. The leading anticancer drug cisplatin, a third generation drug used for colon cancer, oxaliplatin and kiteplatin, promising Pt-based anticancer drugs effective against resistant lines, were administered to transgenic *A. thaliana* plants monitoring their effects on cells from different tissues. The transgenic plants’ cell cytoskeletons were labelled by the green fluorescent protein (GFP)-tagged microtubule-protein TUA6 (TUA6-GFP), while the vacuolar organization was evidenced by two soluble chimerical GFPs (GFPChi and AleuGFP) and one transmembrane GFP-tagged tonoplast intrinsic protein 1-1 (TIP1.1-GFP). The three drugs showed easily recognizable effects on plant subcellular organization, thereby providing evidence for a differentiated drug targeting. Genetically modified *A. thaliana* are confirmed as a possible rapid and low-cost screening tool for better understanding the mechanism of action of human anticancer drugs.

## 1. Introduction

Platinum drugs (cisplatin, *cis*-diamminedichloridoplatinum(II), CDDP; carboplatin, diammine [1,1-cyclobutanedicarboxylato] platinum(II); and oxaliplatin, [(1*R*,2*R*)-cyclohexane-1,2-diamine] (ethanedioato)platinum(II)) are widely used in the clinic and the prototype cisplatin has proven to be effective in the treatment of a variety of tumors such as testicular, ovarian, bladder, head and neck, and small and non-small cell lung cancers [1,2,3,4,5,6,7]. However, resistance and side effects can limit the use of cisplatin [8,9,10,11]. In order to broaden the spectrum of activity and to improve the therapeutic efficacy of cisplatin, carboplatin and oxaliplatin were approved worldwide for clinical use. Oxaliplatin, in particular, contains the 1*R*,2*R*-DACH (DACH, diaminocyclohexane) carrier ligand and currently is one of the most important therapeutic agents used as an adjuvant in the treatment of stage III colon cancer (as part of the FOLFOX, FOLFOXFIRI, and CapeOX chemotherapeutic regimens).

**Figure 1:**
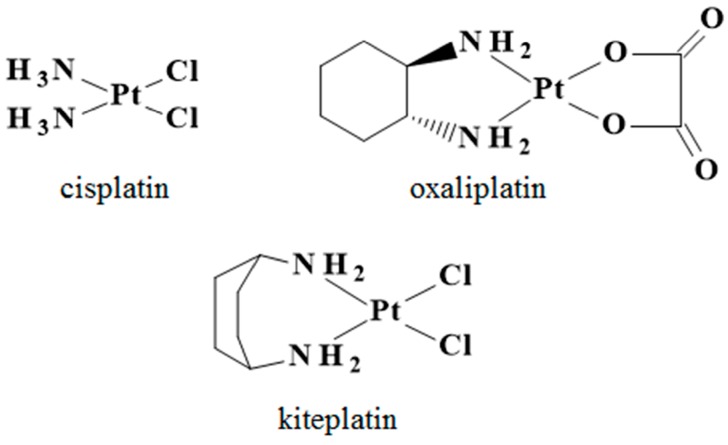
The three platinum-based anticancer compounds investigated in this work.

The compound PtCl_2_(*cis*-1,4-DACH), also dubbed kiteplatin (Figure 1), contains an isomeric form of the oxaliplatin diamine ligand and was introduced into Pt-based drug research many years ago as an alternative to 1*R*,2*R*-DACH Pt-compounds [12]. Notwithstanding some initial interest due to the fact that kiteplatin exhibited better in vitro cytotoxicity than cisplatin and also substantial in vivo activity in L1210 leukemia cell lines resistant to PtCl_2_(1*R*,2*R*-DACH) [13], kiteplatin was set aside, until recently, when it was extensively reinvestigated as a potential new Pt anticancer drug against colorectal cancer including the oxaliplatin-resistant phenotypes [14,15,16,17,18].

The complete evaluation of subcellular effects of new drugs requires a long process, but preliminary screening in vitro provides valid tools to select the most effective candidate molecules. Each model provides information on specific subcellular targets.

DNA is commonly deemed as the primary target of cisplatin effect on cancer cells [9], but the Pt drug is also known to affect the process of tubulin assembly/disassembly [19] and stimulate autophagy [20]. These effects have strong influence on cell vitality beyond DNA replication or repair mechanisms. The negative effect on tubulin assembly/disassembly was proved in vitro. Tubulin does not assemble into microtubules when platinated, producing highly stable and inert circled rings instead [19,21].

Cisplatin, similar to other chemotherapeutic agents, induces cellular and metabolic stress that leads to increased autophagy as a strategy for drug resistance [22,23,24]. Autophagy appears to be part of the immediate response to cisplatin injury, but it also has been shown to be related to cisplatin-induced caspase activation. It was shown in animal cells that when caspase activation begins to increase, autophagy is markedly decreased. Cisplatin-induced activation of caspases targets autophagy proteins for degradation. Autophagy related 5 (Atg5), beclin-1 (Atg6), and Atg12 have been shown to be proteolytically cleaved after cisplatin treatment [20].

In normal conditions, the process is regulated by the concerted action of about 30 evolutionarily conserved autophagy-related Atg proteins. In plants, the mechanisms allowing phagophore generation and its differentiation into autophagosome have not been elucidated in great detail, but they are presumably highly conserved [25,26,27]. Recent studies show that autophagosome biogenesis and vacuolar traffic are closely related [28,29]. It is known that in mammalian cells autophagosome undergoes fusion with endosomes and lysosome to form the amphisome; such a fusion event in plant cells can only be hypothesized as evidenced by preliminary investigations [30]. On the other hand, using *Arabidopsis thaliana* mutants it was shown that endosomal sorting complexes required for transport (ESCRT) components regulating the plant multivesicular bodies morphology play a role in autophagosome formation and subsequent fusion with the vacuole [31]. As a consequence, vacuolar markers, especially those involving the formation of multivesicular bodies, may be used as reporters of autophagic activity.

The multiple targets of Pt drugs demand a specific attention in the design of preliminary screening, promoting the selection of models where several subcellular targets may be monitored in parallel. Largely available transgenic plants can be an example of such models [32].

The conjugation with green fluorescent protein (GFP) can be used to highlight proteins involved in tubulin organization (using TUA6-GFP) [33] and vacuolar transport (using AleuGFP and GFPChi) [32,34]. The GFP-conjugated proteins are suitable markers to monitor the interplay between cisplatin-related compounds and subcellular targets in order to evidence differential biological effects in rapid in vivo preliminary screenings. In this work, we propose the use of transgenic plants expressing GFP-tagged markers as a low-cost platform for the visualization of the effects generated by three Pt anticancer compounds, two already in clinical use, cisplatin and oxaliplatin, and one oxaliplatin analog currently under study, kiteplatin [14,15,16,17,35].

The effect of the three compounds was investigated in transgenic *A. thaliana* plants using a previously reported strategy [32]. The cell cytoskeleton was labelled with GFP-tagged microtubule protein TUA6 (TUA6-GFP) [33] while the vacuolar organization was evidenced by two soluble (AleuGFP and GFPChi) and one transmembrane GFP-tagged tonoplast intrinsic protein 1-1 (TIP1.1-GFP) GFPs. AleuGFP accumulates in the vacuole through COPII (coat protein complex that initiates the budding process from the rough endoplasmic reticulum to the Golgi apparatus)-dependent transport mechanism, GFPChi is sorted through a COPII-independent mechanism and TIP1.1-GFP labels very efficiently all vacuolar membranes [36].

The strong advantage of this approach is that the use of several lines of transgenic plants allows for different subcellular targets to be monitored in parallel, evidencing primary and secondary effects. The screening can warn about the potential detrimental effect of off-target effects or emphasize the beneficial role of multitarget effects.

## 2. Results

### 2.1. Effect of Platinum Compounds on Plant Cell Cytoskeleton

**Figure 2:**
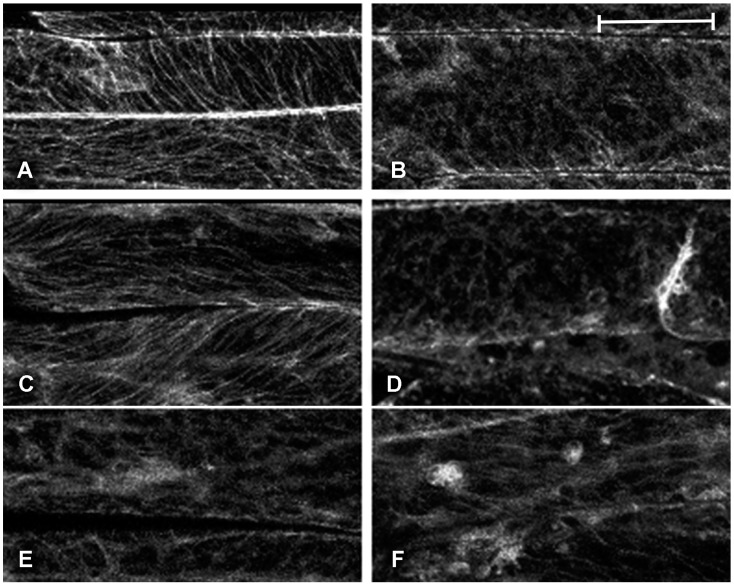
Fluorescent patterns of green fluorescent protein (GFP)-tagged microtubule-protein TUA6 (TUA6-GFP) microtubules marker in elongated petiole cells of transgenic *Arabidopsis* *thaliana*. (**A**) Normal fluorescent pattern of TUA6-GFP as observed after treatment with low drug doses (here 0.1 mg/L, 0.26 µM kiteplatin); (**B**) Perturbed microtubules after treatment with 1 mg/L, 2.5 µM, oxaliplatin; (**C**) Weak perturbation of microtubules orientation after treatment with 5 mg/L, 13 µM, kiteplatin; (**D**) Strong alteration due to treatment with 200 mg/L kiteplatin; (**E**) Clear alteration of TUA6-GFP distribution after treatment with 1 mg/L (3 µM); and (**F**) 200 mg/L (67 µM) cisplatin. Scale bar: 20 µm.

Contrary to the case of cisplatin, which is known to affect cytoskeleton, we have been unable to find specific reports of analogous effects for oxaliplatin and kiteplatin. Platinated tubulin is not able to assemble into microtubules and we planned to monitor this effect by observing the distribution of TUA6-GFP [19]. Also, the reported effect on F-actin polymerization [37] would alter, indirectly, TUA6-GFP distribution. Several doses of the Pt compounds were supplemented to plantlets at increasing concentrations (0.1, 1, 2.5, 5, 10, 30, 50, 100, and 200 mg/L). A high variability in effects was observed starting after 4 h of incubation. However, the observed alterations were occasional and probably due to an increased number of stressed cells rather than a systematic alteration of the cytoskeleton in all cells. The fluorescent pattern in healthy cells remained similar to the normal distribution (Figure 2A). Stressed cells can be induced in all tissues for several reasons, including handling, so that sporadic alteration of fluorescent patterns was not taken into account.

Among the three compounds tested, the strongest effects were observed on the distribution of microtubules after treatment with oxaliplatin was applied; as an example, a strong perturbation of the orientation of microtubules was observed at the concentration of 1 mg/L (2.5 µM) of oxaliplatin (Figure 2B). The effect of kiteplatin was weaker, with microtubules appearing normal with doses up to 5 mg/L (13 µM; Figure 2C) and clear induced alterations only at the highest doses administered (200 mg/L, 0.53 mM; Figure 2D). Cisplatin showed a noticeable effect already at the concentration of 1 mg/L (3.3 µM; Figure 2E), but its effects did not appear to be dose-dependent (Figure 2F).

### 2.2. Effect of Platinum Compounds on Plant Cells’ Central Vacuolar and Golgi Mediated Transport

Interferences with vacuolar transport can modify several aspects of cell compartmentalization. In fact, vacuolar transport in plants has to satisfy different functions, ranging from storage molecules accumulation to homeostasis and degradative catabolic events. Among these processes, some vacuolar transport steps overlap with degradative autophagy since the central vacuole is the final destination of the phagosome [28]. As a consequence, appropriately chosen vacuolar markers can also reveal alterations of autophagy [26]. A reduced or diversified effect on autophagy, generally related to drug resistance, can lead to better and more effective chemotherapics [26].

**Figure 3:**
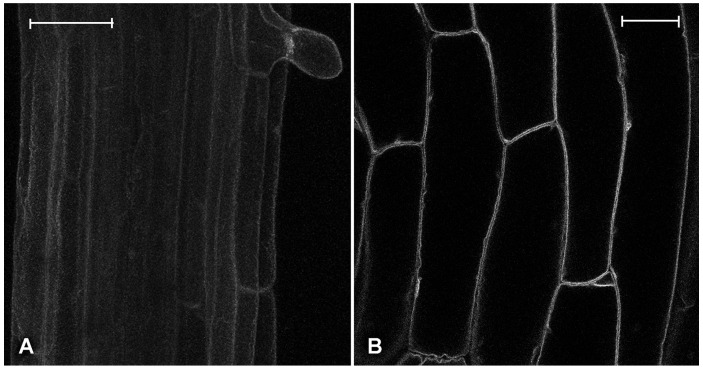
Fluorescent patterns of GFP-tagged tonoplast intrinsic protein 1-1 (TIP1.1-GFP) tonoplast marker, in different tissues. (**A**) Confocal sections projection of the young root at the level of trichoblast differentiation in control conditions; (**B**) Single confocal section of hypocotyl’s cells in control conditions. Scale bar: 20 µm.

At first, we monitored the effect of the Pt compounds on the morphology of the preexisting vacuolar complex observing plantlets expressing the specific membrane aquaporin TIP1.1-GFP [36]. The normal distribution of TIP1.1-GFP on the tonoplast of a unique central vacuole (Figure 3) did not appear altered by the high doses used in our experiments.

**Figure 4:**
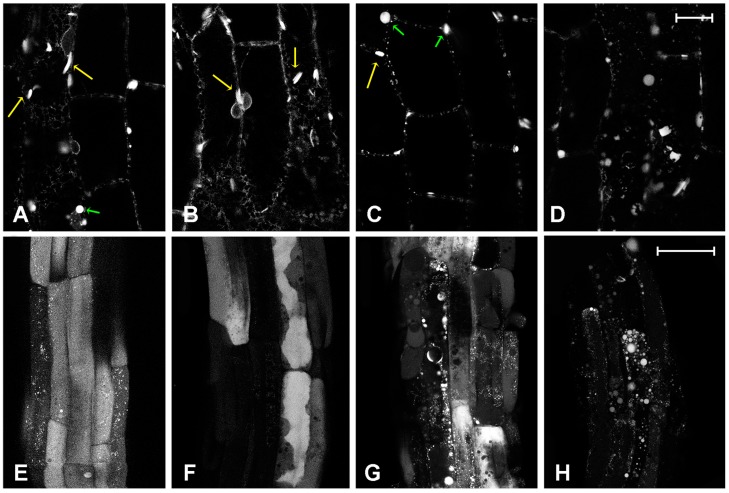
Fluorescent patterns of AleuGFP vacuolar marker, in different tissues. (**A**) Single confocal section of hypocotyl’s cells in control; (**B**) Hypocotyl’s cells treated with 5 mg/L (13 µM) kiteplatin; (**C**) 5 mg/L (13 µM) oxaliplatin or (**D**) 5 mg/L (17 µM) cisplatin. Long yellow arrows indicate endoplasmic reticulum (ER) bodies while short green arrows indicate different large round-shaped compartments. Four confocal sections projection of young root at the end of elongation stage (emerging trichoblasts) in (**E**) control conditions or treated with (**F**) 10 mg/L (26 µM) kiteplatin; (**G**) 10 mg/L (25 µM) oxaliplatin; or (**H**) 10 mg/L (33 µM) cisplatin. Scale bar: 20 µm.

The differential effects on vacuolar transport mechanisms were successfully investigated by observing the different distribution of the fluorescent markers AleuGFP and GFPChi. AleuGFP is used as a marker of the small COPII coat GTPase Sar1-dependent vacuolar traffic and can be associated to the lysosomal traffic of animal cells [38]. With all tested compounds the distribution of AleuGFP in the hypocotyl was altered starting from the dose of 5 mg/L (17, 13, and 12 µM for cisplatin, kiteplatin, and oxaliplatin, respectively; Figure 4B–D). Every drug appeared to affect a different step of the marker traffic. In the control, AleuGFP was distributed among a variety of different compartments: a portion was found in the endoplasmic reticulum (ER), which is the starting compartment for secreted proteins, another in ER bodies with their typical fusiform shape (Figure 4A, long/yellow arrows) [39]. Moreover, AleuGFP was observed in tiny dots, presumably corresponding to pre-vacuolar-compartments (PVCs) and finally in other large round-shaped compartments apparently not integrated in the ER (Figure 4A, green arrows). Treatment with kiteplatin limited the fluorescence to ER and ER bodies (Figure 4B), while oxaliplatin reduced fluorescence in the ER in favor of PVCs and larger round-shaped compartments (Figure 4C). Similar to oxaliplatin, cisplatin reduced ER labelling and caused the formation of more irregular compartments (Figure 4D).

The effect on young roots started to be evident from doses of 10 mg/L (33, 26, and 25 µM for cisplatin, kiteplatin, and oxaliplatin, respectively), again showing differences in the activity of the three Pt-based drugs (Figure 4E–H). Kiteplatin appeared to affect protein synthesis rather than traffic, as evidenced by the absence of fluorescence in the compartments upstream of the vacuole (Figure 4F), but, considering the effect visualized in the hypocotyl, it may also cause the missorting of vacuolar proteins to the apoplast, including proteases. The loss of proteolytic activity in the central vacuole may then explain the persistent GFP fluorescence in this compartment despite the sorting block. Oxaliplatin caused a more evident alteration, in fact the newly formed vacuoles appeared fragmented (Figure 4G). Finally, cisplatin caused an extreme effect, since transport to vacuoles was completely inhibited (Figure 4H). We assume that pre-existing vacuoles were not fragmented by treatment with the drugs since TP1.1-GFP continued to evidence the tonoplast or a unique central vacuole.

### 2.3. Effect of Platinum Compounds on Plant Cell Golgi-Independent Vacuolar Transport

**Figure 5:**
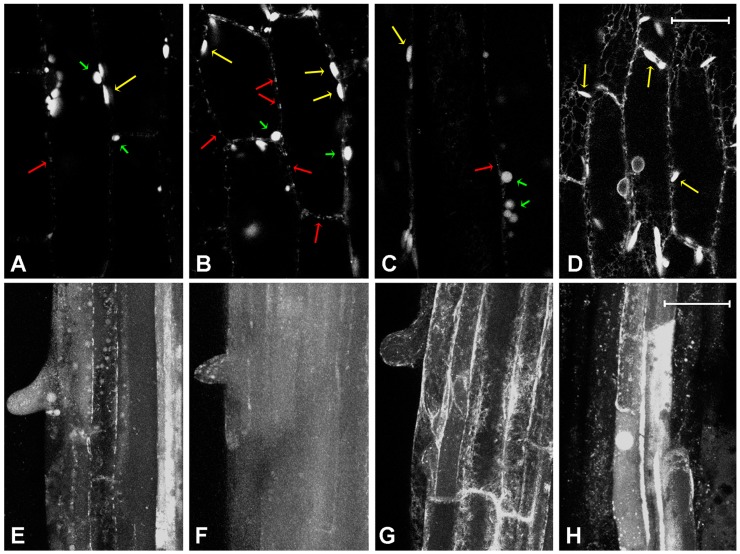
Fluorescent patterns of GFPChi vacuolar marker, in different tissues. Single confocal section of hypocotyl’s cells in (**A**) control; hypocotyl’s cells treated with (**B**) 5 mg/L (13 µM) kiteplatin; (**C**) 5 mg/L (13 µM) oxaliplatin; or (**D**) 5 mg/L (17 µM) cisplatin. Long yellow arrows indicate ER bodies while short green arrows indicate different large round-shaped compartments and long red arrows indicate pre-vacuolar-compartments (PVCs). Four confocal sections projection of young root at the end of elongation stage (emerging trichoblasts) in (**E**) control or (**F**) treated with 10 mg/L (26 µM) kiteplatin; (**G**) 10 mg/L (25 µM) oxaliplatin; or (**H**) 10 mg/L (33 µM) cisplatin. Scale bar: 20 µm.

GFPChi is used as marker of the Sar1-independent vacuolar traffic [38]. This sorting pathway allows proteins to reach the vacuole bypassing the Golgi by way of specific receptors named receptor membrane RING-H2 (RMR) [40], and probably to merge with autophagy derived multi vesicular bodies [28]. The distribution of GFPChi in the hypocotyl was altered by treatment with the Pt drugs starting from the dose of 5 mg/L (Figure 5B–D). Drug effects were less evident in comparison to the experiments where the marker AleuGFP was used (see Figure 5A–D). The distribution of this reporter protein is characterized by the labelling of ER bodies and large pro-vacuolar round-shaped compartments (Figure 5A). Treatment with kiteplatin slightly altered this distribution in two ways: by increasing the number of labelled compartments and by the appearance of some small compartments, similar to PVCs labelled by AleuGFP (Figure 5B). Similar to kiteplatin, oxaliplatin induced a moderate increase of labelled compartments (Figure 5C). Contrary to the case of the two isomeric DACH-platinum drugs, cisplatin caused a clear and typical alteration of the fluorescent pattern, evidenced by the increase of ER labelling in the absence of other membranous compartments (Figure 5D).

Consistent with other markers, the effect on the GFPChi distribution in young root started to be evident from doses of 10 mg/L. The normal pattern showed a transition state, corresponding to expanding cells close to full differentiation, where the central vacuole starts to be labelled while still in presence of small vacuoles (Figure 5E); the effects of the different drugs were clearly distinguishable even if they appeared relatively mild. Kiteplatin promoted the appearance of fluorescence in the central vacuole (Figure 5F); oxaliplatin, on the contrary, caused an apparent block in the ER (Figure 5G), and cisplatin caused an extreme effect whereby it reduced the fluorescence in the central vacuole of root epidermal cells and increased it in cells in the deeper layers, preventing in general the fusion of pro-vacuoles with the central compartment (Figure 5H).

## 3. Discussion

Chemotherapeutic drugs preferentially target proliferating tumor cells, having little or no effect on typically non-replicating healthy cells. The cytoskeleton dynamism, essential for cell division, and autophagy, involved in drugs resistance, are important targets of chemotherapeutic drugs. The molecular targets affecting these mechanisms can also interfere with the endomembrane system in general, generating the risk to observe off-target effects on healthy cells.

New drugs require accurate characterization, but initial screening in vivo can provide useful indications on efficacy and on the risk of off-target effects. Cost-effective screening models are preferable and we recently proposed the use of transgenic plants for this purpose [32]. Any marker labeling the cytoskeleton can reveal its correct or altered organization, but the labeling of the endomembrane system is more problematic. Vacuolar sorting appears to be the most elaborated pathway in the endomembrane system because it involves all compartments, including those playing a role in exocytosis and endocytosis. Recent studies in plants also show that autophagosome biogenesis and vacuolar traffic are closely related [28,29].

Starting from these considerations the monitoring of tubulin organization (TUA6-GFP) and vacuolar transport (AleuGFP and GFPChi [34]) appear to be valid markers to monitor the subcellular targets of cisplatin-related compounds in order to evidence differential biological effects in rapid in vivo preliminary screenings in plants for medical applications. The differences between plant and animal models exist especially among the proteins bridging between the cytoskeleton and the cell membrane [41], but represent a reasonable limit for a cost-effective preliminary screening. In monitoring the processes involving autophagy (such as multi-drugs resistance or apoptosis), the transport of vacuolar markers can be related to dose dependent sensitivity of Atg proteins to the different molecules tested [20,42]. The plant Atg proteins are conserved among eukaryotes and include the animal light chain 3 (LC3) system [43], therefore, the effect on these proteins affects the entire autophagocytotic process. A reduced or diversified effect on autophagy, generally related to drug resistance, can lead to better and more effective chemotherapeutics.

The GFP tagging of α-tubulin TUA6 (TUA6-GFP) allows for monitoring of the status of the cytoskeleton [32] while TIP1.1 GFP tagging (TIP1.1-GFP) is used to monitor the fate of the central vacuole [36], which is already present as a unique central compartment before the treatment. These two markers are very important for interpreting minor effects on the traffic related to membranes and proteins. In fact, cytoskeleton alterations could potentially affect several membrane traffic events while central vacuole reorganization could completely alter the apparent patterns, not necessarily by affecting the traffic mechanism.

Among the three Pt-based drugs tested, the strongest effect on cytoskeleton (Figure 2) was observed in the case of oxaliplatin, with a perturbation of the microtubules orientation starting from the concentration of 1 mg/L (2.5 µM). Also, cisplatin showed a similar effect already at 1 mg/L (3.3 µM), but this drug did not show a clear dose-dependent effect. This may be due to the higher hydrophilicity of cisplatin (log P_o/w_ − 2.27) as compared to kiteplatin (log P_o/w_ − 1.57) and oxaliplatin (log P_o/w_ − 1.39) [14]. Cisplatin may be favored in the penetration of the plant tissue or simply because of the interaction with molecular targets on the cytoskeleton. As a consequence, treatment with 1 mg/L may already be above the saturation dose for such cytoskeleton molecular targets. The goal to evidence differentiated effects for the three drugs was attained, so we did not further investigate the effect of this drug using lower doses.

The effects were particularly visible on newly formed compartments. Experiments with the TIP1.1-GFP marker revealed that the pre-existing central vacuole was not fragmented or significantly altered by treatment with the three drugs. The two soluble vacuolar markers used in this investigation allowed the monitoring of two alternative traffic mechanisms. The N-terminal fusion of the sequence-specific vacuolar sorting determinants (VSD) of the cysteine protease barley Aleurain with GFP (AleuGFP) has been widely used to study vacuolar sorting [44]. AleuGFP is able to reach an acidic vacuole, usually corresponding to the central vacuole [34], and interact with VSR-1 as aleurain itself does [45]. This sorting pathway corresponds to the classic vesicular traffic through the Golgi in animal cells.

In *A. thaliana*, AleuGFP is rapidly exported from the ER to accumulate in small and spherical PVCs before reaching the lytic vacuole where it rapidly loses fluorescence and is degraded [46]. In the hypocotyl of the transgenic plants considered, these steps are evident because fluorescence is distributed between ER, ER bodies with a typical fusiform shape, tiny dots presumably corresponding to PVCs, and other large round-shaped compartments (the central vacuole is not fluorescent because of degradation) [34,46]. Kiteplatin affected ER export; in fact, the fluorescence was retained in the ER and ER bodies. Oxaliplatin had a different effect because it reduced fluorescence in the ER in favor of PVCs and larger round-shaped compartments. Cisplatin showed an effect similar to that of oxaliplatin in reducing ER labelling and causing the formation of more irregular compartments.

The effect on the young roots started to be evident from higher doses (10 mg/L), being more difficult to be detected. The central vacuole (i.e., the final destination of the marker) was fluorescent and no other elements were visible in the pattern. Nonetheless, it is possible to observe differences and provide an interpretation of the data: kiteplatin appeared to affect protein synthesis rather than protein traffic because the compartments upstream of the vacuole appeared to have lost their fluorescence. Oxaliplatin caused a more evident alteration because newly formed vacuoles appeared fragmented and, finally, cisplatin caused an even more extreme effect because transport to vacuoles was completely inhibited and fluorescence of the central vacuole was lost in favor of aberrant compartments that are not a fragmentation of the pre-existing vacuole (as shown by the experiments with TIP1.1-GFP, Figure 3), but aberrant intermediate compartments (pro-vacuoles) disturbed in their maturation process.

GFPChi and other reporters exposing the C-terminal signal from chitinase A are emerging as potential markers for the direct transport from ER to plant vacuole [44], a pathway closely related to the autophagic process [30,31]. It is possible to observe the autophagy marker ATG8f labelling colocalize with GFPChi fluorescence in pro-vacuoles (Figure S1). Moreover the intracellular sorting driven by the chitinase A signal is sensitive to wortmannin, which is also an autophagy inhibitor [47]. This fungal metabolite irreversibly inhibits phosphatidylinositol 3-kinase (PI3K).

Despite controversies [48], it is known that autophagic activity plays an important role in the progression of malignant tumors [49], and also the molecular signaling pathway of PI3K is known to be involved in tumor proliferation, apoptosis, metastasis, etc. [49]. Altered PI3K activity can facilitate apoptosis, thus inhibiting the tumor growth, and drugs targeting the inhibition of PI3K pathway are receiving growing attention in antitumor drug research.

The drugs’ effect on GFPChi in the hypocotyl was less evident than on AleuGFP distribution. Both kiteplatin and oxaliplatin caused the increase of labelled compartments. Only cisplatin caused a clear and typical alteration of fluorescent pattern with the increase of ER labelling in the absence of downstream compartments.

The effect on the GFPChi distribution in young roots was more informative. This is due to the fact that the normal pattern of GFPChi in the young root is visualized in the expansion area. In this area, it is evidently a transition state where the central vacuole starts to be labelled but small pro-vacuoles are still visible. The effects of the different platinum drugs were clearly distinguishable in this area of the root even if they appeared relatively mild: kiteplatin appeared to promote the accumulation of fluorescence in the central vacuole, oxaliplatin caused an apparent block in the ER and, finally, cisplatin caused the strongest effect by preventing the fusion of pro-vacuoles with the central compartment, acting especially in the root epidermal cells usually characterized by a more homogenous pattern.

In the present paper, we reported additional applications of transgenic *A. thaliana* plants as a preliminary screening tool to evaluate new antitumor drugs. If the further characterization of these effects will find confirmation in comparative studies in animal cells, the approach may be validated as a cost-effective method. The plants were used to pre-screen both clinically approved and experimental platinum-based drugs by analyzing their effects on the subcellular compartments.

Chemotherapeutics interfere with cell vitality, affecting different metabolic and traffic pathways, influencing cell compartmentalization such as autophagy. This could act as a survival mechanism, although in some cases is able to trigger cell necrosis [50]. In fact, it was shown that upregulation of autophagy can remarkably reduce the sensitivity to treatment of osteosarcoma cells to cisplatin-based chemotherapy [51]. As a consequence, autophagy inhibition could also significantly enhance the sensitivity of tumor cells to chemotherapy [52]. Of course, a detailed characterization of Pt drugs subcellular effects, including the autophagic process, is not possible using these markers. The vacuolar markers were selected to label the entire secretory pathway and its compartments and not the autophagic compartments in particular. The GFPChi sorting is expected to be influenced by autophagy defects because of the cross-talk of the two mechanisms, but more focused studies would require more specific markers. At the moment, we know that the autophagy marker Atg8f tagged with red fluorescent protein (RFP), after the formation of small phagosomes, merge to GFPChi labeled pro-vacuoles (Figure S1). In the future we plan to expand screening studies producing double transgenic plants expressing at once TUA6-GFP and RFP-based markers for the vacuole (RFPChi) and the autophagosome (RFP-Atg8f [26]).

The information we derived from the vacuolar markers is innovative and, unfortunately, we do not have analog experimental models for comparison. Hence, our conclusions were based on the implicit assumption that cellular targets are conserved in plants and animals. At any rate, we observed differentiated effects of the three drugs on endomembranes, which were independent from the drugs’ effect on cytoskeleton, or at least without evident correlation. The effects on endomembranes could be considered off-target.

Experiments showed that oxaliplatin perturbs several cellular compartments, namely ER export and Golgi transport, with effects similar to cisplatin. This indicates several undesirable toxic effects, while kiteplatin has a visibly stronger effect on GFPChi distribution, suggesting a potentially specific effect on the autophagic process. Such undesirable toxic effects, not restricted to the cytoskeleton, would affect tumor as well as healthy cells. In some cases, when affecting autophagy, off-target effects may also have positive consequences. For example, the interference with autophagy could be associated with kiteplatin efficacy against oxaliplatin-resistant colorectal cancer [14], and, if further confirmed in mammalian tumor cells, could provide a tool to select chemotherapeutics to overcome drug resistance in cell lines exploiting such a specific mechanism.

Further developing transgenic plants as screening tools for new drugs appears to be a promising investment for the preliminary investigation of specific and non-specific, as well as off-target, effects.

## 4. Materials and Methods

### 4.1. Chemicals

Cisplatin (MW 300.1) [53], oxaliplatin (MW 397.29) [54], and kiteplatin (MW 380.17) [32] were synthesized according to published methods.

### 4.2. Transgenic Plants and Confocal Microscopy

Four chimerical GFP-tagged markers were stably expressed in *A. thaliana*. GFP-tagged α-tubulin TUA6 (TUA6-GFP) was expressed in transgenic *A. thaliana* ecotype Columbia under the control of the CaMV 35S promoter [33]. Two soluble GFPs (AleuGFP and GFPChi) differently sorted in the endomembrane system [34], and in addition the membrane aquaporin TIP1.1 tagged with GFP (TIP1.1-GFP) [36] was expressed in transgenic *A. thaliana* cv. Wassilewskaja under the control of the CaMV 35S promoter.

Transgenic plantlets were grown from T2 seeds on sterile solid Murashige and Skoog basal medium (MS, 3% sucrose, 0.8% agar) under continuous light (25 µmol·m^−2^·s^−1^) at 23 °C. Observed samples consisted of plantlets transferred to liquid medium, supplemented with drugs, into multiwell plates 10 days after germination and monitored in the following 24–36 h.

Full plantlets or primary leaves, including petioles, were mounted for microscopic observation in water under glass coverslips and examined using a confocal laser-microscope LSM 710 Zeiss (ZEN Software, GmbH, Jena, Germany). GFP markers were detected in the wavelength range 505–530 nm. Excitation wavelength of 488 nm was used. For the observation reported in the supplemental figure, the chlorophyll fluorescence excited with the same wavelength of 488 nm was detected above 650 nm. The excitation of RFP was obtained with the laser at 543 nm and emission was detected between 590–620 nm.

## Supplementary Materials

Supplementary materials can be found at www.mdpi.com/1422-0067/18/2/306/s1.

## Abbreviations

DACH	Diaminocyclohexane
Atg	Autophagy related proteins
GFP	Green fluorescent protein
COPII	Coat protein complex that initiates the budding process from the rough endoplasmic reticulum to the Golgi apparatus
ER	Endoplasmic reticulum
PI3K	Phosphatidylinositol 3-kinase
