# Supplementary material for: Cisplatin, Oxaliplatin, and Kiteplatin Subcellular Effects Compared in a Plant Model
Source: Int J Mol Sci. 2017 Jan 31;18(2):306. doi: 10.3390/ijms18020306 (PMC5343842; doi:10.3390/ijms18020306)
Supplement: Supplementary file 1 [file ijms-18-00306-s001.pdf]

Supplementary Materials: Cisplatin, Oxaliplatin, and Kiteplatin Subcellular Effects Compared in a Plant Model

Paride Papadia, Fabrizio Barozzi, James D. Hoeschele, Gabriella Piro, Nicola Margiotta and Gian-Pietro Di Sansebastiano

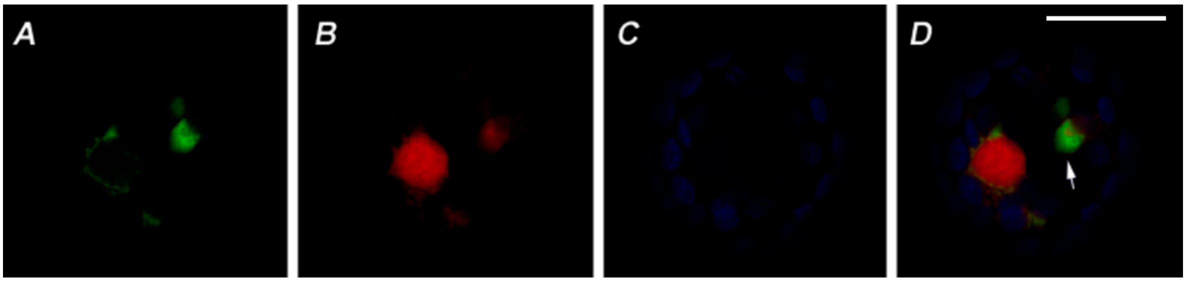

Figure S1. Transient transformation of *Nicotiana tabacum* protoplasts allows the rapid visualization of proteins distribution thanks to fluorescent tags. The method is routinely used and was previously described [1]. The constructs here co-expressed for the first time, were described elsewhere (GFPChi [2] and RFP-Atg8f [3]). *N. tabacum* protoplast transiently expressing the vacuolar GFPChi emitting green fluorescence (**A**) and the autophagy marker RFP-Atg8f emitting red fluorescence (**B**). The chlorophyll autofluorescence is represented in blue (**C**). GFPChi label perinuclear ER and small compartments, RFP-Atg8f fluorescence is diffused in the nucleus and soluble in the cytosol when not recruited on the phagophore, but the two overlap in the pro-vacuoles (or small vacuoles) characteristic of GFPChi sorting (**D**, arrow). Scale bar: 20 μm.

References

1. De Benedictis, M.; Bleve, G.; Faraco, M.; Stigliano, E.; Grieco, F.; Piro, G.; Dalessandro, G.; Di Sansebastiano, G.P. AtSYP51/52 Functions Diverge in the Post-Golgi Traffic and Differently Affect Vacuolar Sorting. *Mol. Plant.* **2013**, *6*, 916–930.
2. Di Sansebastiano, G.P.; Paris, N.; Marc-Martin, S.; Neuhaus, J.-M. Regeneration of a lytic central vacuole and of neutral peripheral vacuoles can be visualized by green fluorescent proteins targeted to either type of vacuoles. *Plant Physiol.* **2001**, *126*, 78–86.
3. Honig, A.; Avin-Wittenberg, T.; Ufaz, S.; Galili, G. A new type of compartment, defined by plant-specific Atg8-interacting proteins, is induced upon exposure of Arabidopsis plants to carbon starvation. *Plant Cell.* **2012**, *24*, 288–303.
